# Transcriptome analysis revealed the dynamic oil accumulation in *Symplocos paniculata* fruit

**DOI:** 10.1186/s12864-016-3275-0

**Published:** 2016-11-16

**Authors:** Qiang Liu, Youping Sun, Jinzheng Chen, Peiwang Li, Changzhu Li, Genhua Niu, Lijuan Jiang

**Affiliations:** 1 Central South University of Forestry and Technology, 498 South Shaoshan Rd., Changsha, Hunan 410004 China; 2 Hunan Academy of Forestry, 658 South Shaoshan Rd., Changsha, Hunan 410004 China; 3 Texas A&M AgriLife Research Center at El Paso, 1380 A&M Circle, El Paso, TX 79927 USA

**Keywords:** Differentially expressed profile, Oil accumulation, *Symplocos paniculata*, Transcriptome analysis

## Abstract

**Background:**

*Symplocos paniculata*, asiatic sweetleaf or sapphire berry, is a widespread shrub or small tree from *Symplocaceae* with high oil content and excellent fatty acid composition in fruit. It has been used as feedstocks for biodiesel and cooking oil production in China. Little transcriptome information is available on the regulatory molecular mechanism of oil accumulation at different fruit development stages.

**Results:**

The transcriptome at four different stages of fruit development (10, 80,140, and 170 days after flowering) of *S. paniculata* were analyzed. Approximately 28 million high quality clean reads were generated. These reads were trimmed and assembled into 182,904 non-redundant putative transcripts with a mean length of 592.91 bp and N50 length of 785 bp, respectively. Based on the functional annotation through Basic Local Alignment Search Tool (BLAST) with public protein database, the key enzymes involved in lipid metabolism were identified, and a schematic diagram of the pathway and temporal expression patterns of lipid metabolism was established. About 13,939 differentially expressed unigenes (DEGs) were screened out using differentially expressed sequencing (DESeq) method. The transcriptional regulatory patterns of the identified enzymes were highly related to the dynamic oil accumulation along with the fruit development of *S. paniculata*. In addition, quantitative real-time PCR (qRT-PCR) of six vital genes was significantly correlated with DESeq data.

**Conclusions:**

The transcriptome sequences obtained and deposited in NCBI would enrich the public database and provide an unprecedented resource for the discovery of the genes associated with lipid metabolism pathway in *S. paniculata*. Results in this study will lay the foundation for exploring transcriptional regulatory profiles, elucidating molecular regulatory mechanisms, and accelerating genetic engineering process to improve the yield and quality of seed oil of *S. paniculata*.

**Electronic supplementary material:**

The online version of this article (doi:10.1186/s12864-016-3275-0) contains supplementary material, which is available to authorized users.

## Background

*Symplocos paniculata*, a member of *Symplocaceae*, is a woody oil plant native to China with notable ecological and economic importance [1]. *S. paniculata* has a high adaptability to different temperature zones and varying soil conditions. It grows well in barren, salty, and severe drought soil like marginal land and arid areas [2]. A mature tree can yield up to 20 kg fruit [3]. The whole fruit contains 36.6% oil [4], of which 79.8% is unsaturated fatty acid. Due to high fruit yield and oil content, *S. paniculata* serves as an ideal feedstock for bio-diesel and edible oil production [5, 6]. *S. paniculata* also has other industrial uses such as ink surfactants, lubricants, and soap. However, oil production from *S. paniculata* fruits is still limited. There is an urgent demand for developing novel cultivars with improved oil yield and quality for biodiesel and edible oil production.

Like avocado, oil palm, and olive, *S. paniculata* accumulates copious amount of oil in both seed and mesocarp of the fruit [7]. Oil is mainly stored as triacylglycerols (TAG) in oil bodies in seed or in oil cells in the mesocarp of the fruit [3, 8]. The oil yield and quality in developing fruits is regulated by a number of enzymes that take part in lipid biosynthesis. Lipid biosynthesis consists of fatty acid synthesis and TAG assembly at multiple subcellular organelles, and its transcriptional regulatory patterns varied with plant species [9]. Previous studies reported genetic manipulation has been used to increase TAG yield and quality (i.e., fatty acid composition) [10]. However, the overall expression and regulation profiles of genes involved in the lipid biosynthesis of *S. paniculata* still remain unclear. It is essential to identify key genes that are related to lipid biosynthesis in the fruit development of *S. paniculata*. If such genes are identified, molecular breeding would increase the fruit oil content and improve fatty acid composition.

RNA-Seq is one of the next generation sequencing technologies that has developed in recent years and created unprecedented opportunities for generating genomic or transcriptomic information. It is widely used for exploring functional genes [11], constructing expression and transcriptional regulatory profiles [12], discovering molecular markers such as simple sequence repeats (SSRs) and single nucleotide polymorphisms (SNPs) [13, 14], and investigating comparative and evolutionary genomics [15, 16]. This technique is efficient to generate a large amount of genetic data. Since the generated reads can assemble without a reference genome, it is also an ideal tool for transcriptome sequencing for those species without a sequenced genome [17]. Recently, RNA-Seq has been used to analyze the transcriptomic profiles of oil accumulation in oil plants such as Jatropha (*Jatropha curcas*) [18], oil tea (*Camellia oleifera*) [19], oil palm (*Elaeis guineensis*) [20], peanut (*Arachis hypogaea*) [21], rapeseed (*Brassica napus*) [22], and sesame (*Sesamum indicum*) [23]. However, the functional genes involved in the oil biosynthesis and metabolism of *S. paniculata* are not yet to be investigated.

In our study, transcriptome analysis of *S. paniculata* was conducted using Illumina high-throughput sequencing platforms 2000. RNA extracted from fresh fruits at four different development stages (10, 80, 140, and 170 days after flowering) was pooled as a sample to establish a cDNA library. The objectives of this study were to: (1) investigate the functional unigenes encoding vital enzymes associated with lipid biosynthesis and metabolism; (2) identify the annotation of complete transcriptome to the functional unigenes; (3) reconstruct the fatty acid (FA) and triacylglycerol (TAG) pathways using the identified enzymes; (4) determine the up- or down-regulated enzymes through the comparison of gene expression at different stages of oil accumulation; and (5) validate the key genes through quantitative real-time PCR (qRT-PCR). These results would help enrich the public database with a large number of sequences. They also benefit breeding efforts in increasing oil content, in modifying fatty acid composition, and in other target characteristics using genetic engineering approaches.

## Methods

### Plant materials and RNA extraction

The fruits of *S. paniculata* from accession C3 were collected every 10 days after flowering (DAF) in 2013 from the experimental station at Hunan Academy of Forestry, Changsha, Hunan, China (28°07′10.38″ N, 113°02′53.16″E, and 94.5 m). These sampling was discontinued at 170 DAF because fruits started to shed. The fruit oil content was determined according to Chinese national standard methods GB/T 5512-2008 [3] with a SZE-101Fat Analyzer (Shanghai Shine Jan Instruments Co. Ltd., Shanghai, China). In brief, fruits were dried at 70 °C for 3 days, and approximately 4 ~ 5 g of fruits were grounded into powder. Powder samples were weighed (w_0_, g) and extracted in a petroleum ether (99.7%, boiling point range 30 to 60 °C) as solvent at 62.5 °C for 6 hours. The residual was dried at 105 °C in vacuum for 2 hours and weighted (w_1_, g). The total fruit oil content (%) was calculated as follows: % = (w_0_-w_1_)/w_0_ × 100%.

Fatty acid components of the fruit oil was analyzed using Clarus 600 gas chromatograph-mass spectrometer (GC-MS, Perkin Elmer Instrument Co., Ltd, Shanghai, China). The samples were saponified first and then injected into a free fatty acid polyester (FFAP) column (0.3 mm × 25 m). Oven temperature was programmed as follows: held at 40 °C for 1 min, increased to 100 °C at 20 °C per minute and held at 100 °C for 2 min, increased to 220 °C at 20 °C per minute and held at 220 °C for 2 min, increased to 280 °C at 20 °C per minute and held at 280 °C for 5 min. Carrier gas, helium (He), was provided at a flow rate of 1.1 per minute in the column. The injector temperature was 250 °C, and the injection volume was 1 μL. GC and MS interface temperature and ion source temperature was 270 and 230 °C, respectively. Electron impact energy of mass spectrometer was 70 eV, and scanning quality ranged from 15 to 500 amu. The fatty acid components were identified using the Wiley mass spectral library [24]. Relative percentage of fatty acid compositions was determined on the basis of the peak areas.

According to the dynamic pattern of oil accumulation, the fresh fruits at four representative developmental stages (10, 80, 140, and 170 DAF) from the same tree were selected as the experimental materials for transcriptomic analysis (10 DAF as a control). Fresh fruits were removed from the mother tree and frozen immediately in liquid nitrogen and stored at −80 °C for RNA extraction.

Fresh fruits (1 ~ 2 g) from each time-point were then grounded into powder using liquid nitrogen. Total RNA was isolated and purified separately according to the manufacturer’s protocol using the Spin Column Plant Total RNA Purification kit (Sangon Biotech, Co. Ltd. Shanghai, China). Extracted RNA was quantified using a Nanodrop ND-2000 spectrophotometer (Nanodrop Technologies, Inc., Wilmington, DE, USA) and Agilent Bioanalyzer 2100 system (Agilent Technologies, Santa Clara, CA, USA). The 260/280 nm ratio of samples ranged from 1.9 to 2.1, and the average RNA integrity number (RIN) was over 8. All the results showed that the extracted RNA was of high quality without any apparent degradation and was suitable for further cDNA synthesis and RNA-Seq.

### cDNA library construction and illumina sequencing

The cDNA library was constructed from a mixed RNA pool using Illumina TruSeq RNA Sample Preparation kit (Illumina, Inc., San Diego, CA, USA). According to the manufacturer’s recommendations, the poly-(A) mRNA was isolated from the total RNA using oligo (dT) beads. The mRNA was chopped into short fragments using a fragmentation buffer. The first-strand cDNA was generated by reverse transcription using a random hexamer-prime, whereas the second strand of cDNA was synthesized with the ligation of the adaptor including DNA polymerase I and RNase H. Subsequently, the cDNA fragments of approximately 200 bp in length were selected using gel electrophoresis and amplified through 15-cycle PCR. Enriched cDNA fragments were purified and quantified using Agilent Bioanalyzer 2100 system.

The cDNA library was sequenced on the high-throughput Illumina Sequencing platform (HiSeq 2000, Illumina, Inc., San Diego, CA, USA). All adapter sequences including low-quality sequences (reads with ambiguous bases ‘N’) and reads with more than 10% Q20 bases were filtered out of data (raw reads), and the remaining reads were denoted as clean reads. All clean reads were assembled into unigenes with Trinity software (version 2014-04-13, Broad Institute of MIT and Harvard, Cambridge, MA, USA) [25]. All high-quality reads were deposited in the National Center for Biotechnology Information (NCBI) Short Read Archive (SRA) database (http://www.ncbi.nlm.nih.gov/sra).

### Functional unigenes annotation

Assembled unigenes were annotated using BLAST alignment with an E value threshold of 10^−5^ against the following four public protein databases: Non-redundant (NR) protein database, Gene Ontology (GO) protein database [26], Clusters of Orthologous Groups (COGs) protein database [27], and Kyoto Encyclopedia of Genes and Genomes (KEGG) protein database [28]. Each assembled sequence was given a gene name based on the best BLAST hit (highest score). But this search was limited to the first 10 significant hits for each query in order to increase the computational speed. The Open Reading Frame (ORF) of the unigenes without BLAST hits were predicted using ESTscan (version 3.0.3) [29]. The best BLAST hit from the NR database for each transcript was submitted to BLAST2GO platform (version 2.5) (http://www.blast2go.org/) to retrieve GO terms for each unigene based on the relationship between gene names and GO terms. EC number was also assigned and passed based on the BLAST2GO results. Moreover, the sequences with designated ECs obtained from BLAST2GO were mapped to the KEGG metabolic pathway database to understand the functional unigenes involved in metabolic pathways. KEGG Automatic Annotation Server (KAAS) [30] and KEGG Orthology Based Annotation System (KOBAS) (version 2.0) [31] were used to automatically annotate the unigenes that code for known orthologues of plant enzymes involved in fatty acid biosynthesis, fatty acid degradation, TAG biosynthesis, and TAG degradation pathways.

### Differential expression of unigenes

Differentially expressed unigenes (DEGs) were screened using differentially expressed sequencing (DESeq) method [32]. The unigenes expression levels were statistically calculated using reads per kilobase transcriptome per million mapped reads (RPKM) [33]. The RPKM method was used to normalize the abundances of transcripts to eliminate the influence of different gene lengths and sequencing discrepancies on the gene expression calculation. A 3-fold difference of RPKM was used to identify the genes differentially expressed between two developmental stages. And DEGs were functional annotated in GO and KEGG database.

### Quantitative RT-PCR validation

Six unigenes involved in different lipid metabolism pathways with different regulation modes were selected for the validation using real time qPCR. The gene-specific primer pairs were designed using Primer Premier 5.0 software (Premier Biosoft International, Palo Alto, CA, USA). Total RNA was isolated from fruits sampled at 10, 80, 140, and 170 DAF as the description mentioned above. cDNA was synthesized using the SYBR Premix Ex Taq Kit (TaKaRa, Mountain View, CA, USA) according to the manufacturer’s protocol. Relative mRNA abundance of the selected genes was determined using Multicolor Real-Time PCR Detection System (Bio-Rad, Hercules, CA, USA). The actin-related protein 3 (ACTR3) and 18S rRNA were chosen as an internal control for normalization. The conditions for all reactions were 95 °C for 2 min, 40 cycles of 95 °C for 15 s, followed by 60 °C for 15 s, and 95 °C for 15 s. Three technical repetitions were performed for qRT-PCR. The relative expression level of qPCR results for each unigene was calculated using the comparative cycle threshold (ΔΔCt) method [34]. Correlation analysis between RPKM with ΔΔCt was performed using JMP (Version 12, SAS Institute Inc., Cary, NC).

## Results and discussion

### Temporal pattern of oil accumulation and fatty acid compositions

The temporal pattern of oil accumulation and fatty acid compositions in the fruit of *S. paniculata* at four different development stages were investigated. There was a little oil accumulated (0.23 ~ 2.56%) in the fruit of *S. paniculata* from 10 to 80 DAF, but a most noticeable change in the fruit oil content happened during the period of 80 to 140 DAF, when an average of 0.5% oil accumulated every day. Thereafter, the oil content still gradually increased till 170 DAF when the maximum fruit oil content (36.6%) was reached (Additional file 1: Figure S1). The oil content of *S. paniculata* fruit might keep increasing after 170 ADF, however, its fruits started to shed at 170 DAF and the sampling was discontinued. Therefore, the oil content at 170 ADF was determined as the maximum oil content during the fruit developmental stages. The fruit oil was mainly composed of saturated fatty acid such as palmitic acid (C16:0) and stearic acid (C18:0) and unsaturated fatty acid such as oleic acid (C18:1), linoleic acid (C18:2), and linolenic acid (C18:3). Oleic acid (C18:1) was the major component of the fruit oil, and up to 50.6% of C18:1 accumulated at 90 days after blooming. Linoleic acid (C18:2) decreased from 10 to 90 DAF and then increased along with the maturity of fruit. However, other fatty acids such as linolenic acid (C18:3), palmitic acid (C16:0), and stearic acid (C18:0) decreased throughout the fruit development (Additional file 2: Figure S2). Fresh fruits at 10, 80, 140, and 170 DAF, representing four different development stages, were selected for further transcriptomic analysis.

**Fig. 1:**
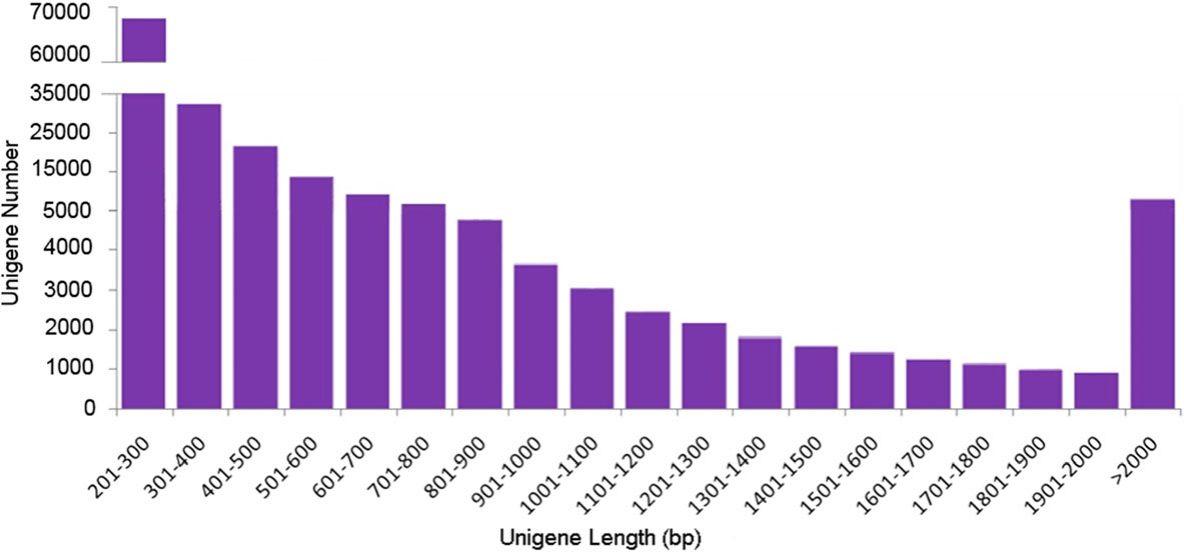
Frequency of *Symplocos paniculata* unigenes

Total RNA were extracted from the fresh fruits at four different development stages of *S. paniculata* and used to construct four cDNA libraries separately. The cDNA libraries consisted of a total of 29,648,536 raw reads with an average length of 548 bp. After the adaptor and low quality reads were removed, approximately 27,827,593 (93.86%) high quality clean reads were obtained. The average read size, Q20 percentage (sequencing error rate, 0.04%), and GC percentage for each library was 537 bp, 95.87, and 46.04%, respectively (Additional file 3: Table S1). All clean reads were mutually aligned and assembled using Trinity software (version 2014-04-13, Broad Institute of MIT and Harvard, Cambridge, MA, USA). A total of 218,425 contigs with a mean length of 680 bp was obtained. Then a final contigs assembly produced 182,904 non-redundant unigenes with an average length of 593 bp and N50 length of 785 bp (Table 1). The length of unigenes’ sequences mainly ranged from 200 to 2,000 nt, and unigene number gradually decreased without obvious disjunction as the length of sequences increased. These results indicate that a good continuity and high quality of RNA sequencing was conducted (Fig. 1). Of the unigenes, 67,729 (37%) were short sequences between 200 nt and 300 nt, whereas only 7,652 (0.04%) were longer than 2,000 nt. These results indicated short sequences dominated the unigenes of *S. paniculata*. All high-quality reads were deposited in the National Center for Biotechnology Information (NCBI) Short Read Archive (SRA) database under accession numbers PRJNA312748. Currently, a complete genome of *S. paniculata* does not exist in such a public database. Therefore, the transcriptome data set reported in this paper would enrich the database for future functional genes annotation, key enzyme identification, and genetic differentiation expression analysis*.*

**Table 1:** Summary of the sequencing data of *Symplocos paniculata*

	Total number	Total length (bp)	Mean length (bp)	N50 (bp)
Contigs	218,425	148,553,601	680.11	1,031
Unigenes	182,904	108,445,745	592.91	785

### Functional annotation of non-redundant unigenes

Based on the predicted sequences from a final contigs assembly, the 182,904 unigenes were annotated using the BLAST program against NR, GO, COG, KEGG protein databases. A total of 67,202 (36.74%), 25,893 (14.16%), 26,831 (14.67%), and 31,407 (17.17%) unigenes had the most significant BLAST matches with known proteins in the NR, GO, COGs, and KEGG database, respectively. Of the unigenes, 67,379 (36.84%) had the best BLAST matches in at least one of the four databases, whereas 10,306 (5.63%) had the best BLAST matches to proteins in all of the four databases (Table 2). However, the remaining 115,525 (63.16%) unigenes had no significant annotation hit, which may result from a large number of short sequences (67,729, 37%) generated in our study. The short sequences do not have a characterized protein domain and may cause the false-negative results [35]. Similar results were seen in previous investigations on yellow horn species [36].

**Table 2:** Functional annotation of *Symplocos paniculata* unigenes in public protein databases

	Number of unigenes	Percentage (%)
Annotated in NR	67,202	36.74
Annotated in GO	25,893	14.16
Annotated in COGs	26,831	14.67
Annotated in KEGG	31,407	17.17
Annotated in all four databases	10,306	5.63
Annotated in at least one of four databases	67,379	36.84

**Fig. 2:**
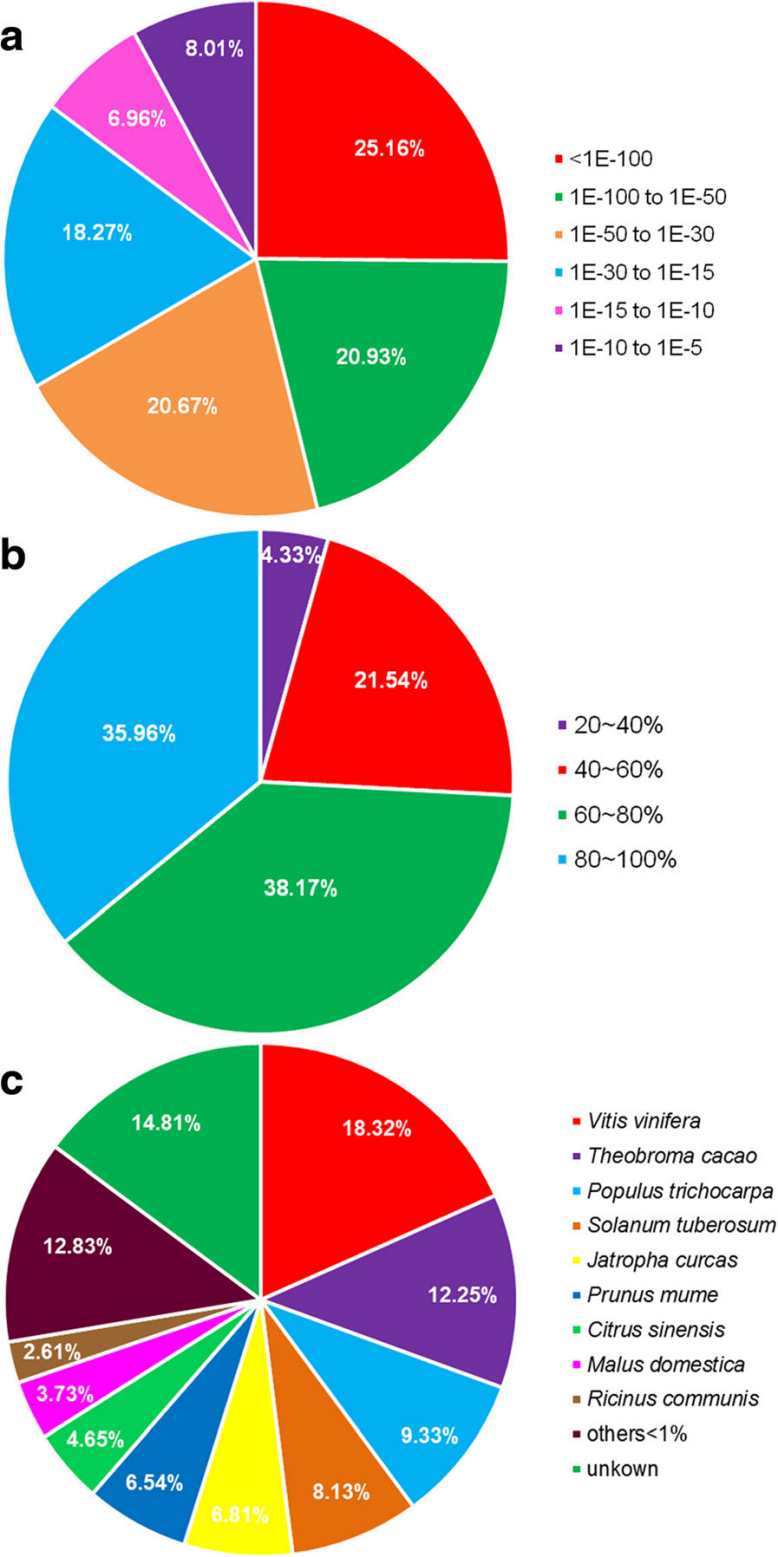
Similarity analysis between *Symplocos paniculata* unigenes and NR database. **a** E-value (<1E-5) distribution of top BLAST hits for each *S. paniculata* unigene, **b** Similarity of *S. paniculata* putative proteins with known proteins in NR database (>20%), **c** Top-hit species distribution for BLAST matches for *S. paniculata* unigenes

The similarity analysis between *S. paniculata* unigenes and NR protein databases was conducted using BLAST matches (Fig. 2). The percentage of putative proteins was 25.16 20.93 20.67 and 18.27% at the four categories of low E-value (Fig. 2a). There were 21.54 38.17 and 35.96% putative proteins showing 40 ~ 60%, 60 ~ 68%, 80 ~ 100% of similarity, respectively, with the known proteins in NR protein database (Fig. 2b). These results ensured the accuracy and reliability of BLAST match analyses. *S. paniculata* unigenes had significant matches with homology genes from grape (*Vitis vinifera*) (12,314, 18.32%), followed by *Theobroma cacao* (8,232, 12.25%), black cottonwood (*Populus trichocarpa*) (6,270, 9.33%), potato (*Solanum tuberosum*) (5464, 8.13%), Jatropha (4,576, 6.81%), Japanese apricot (*Prunus mume*) (4395, 6.54%), orange (*Citrus sinensis*) (3,125, 4.65%), apple (*Malus domestica*) (2,507, 3.73%), castorbean (*Ricinus communis*) (1,754, 2.61%) and other species (8,622,12.83%). In addition, there were 9,946 (14.81%) unigenes that were not homology to any genes of any plant species (Fig. 2c).

**Fig. 3:**
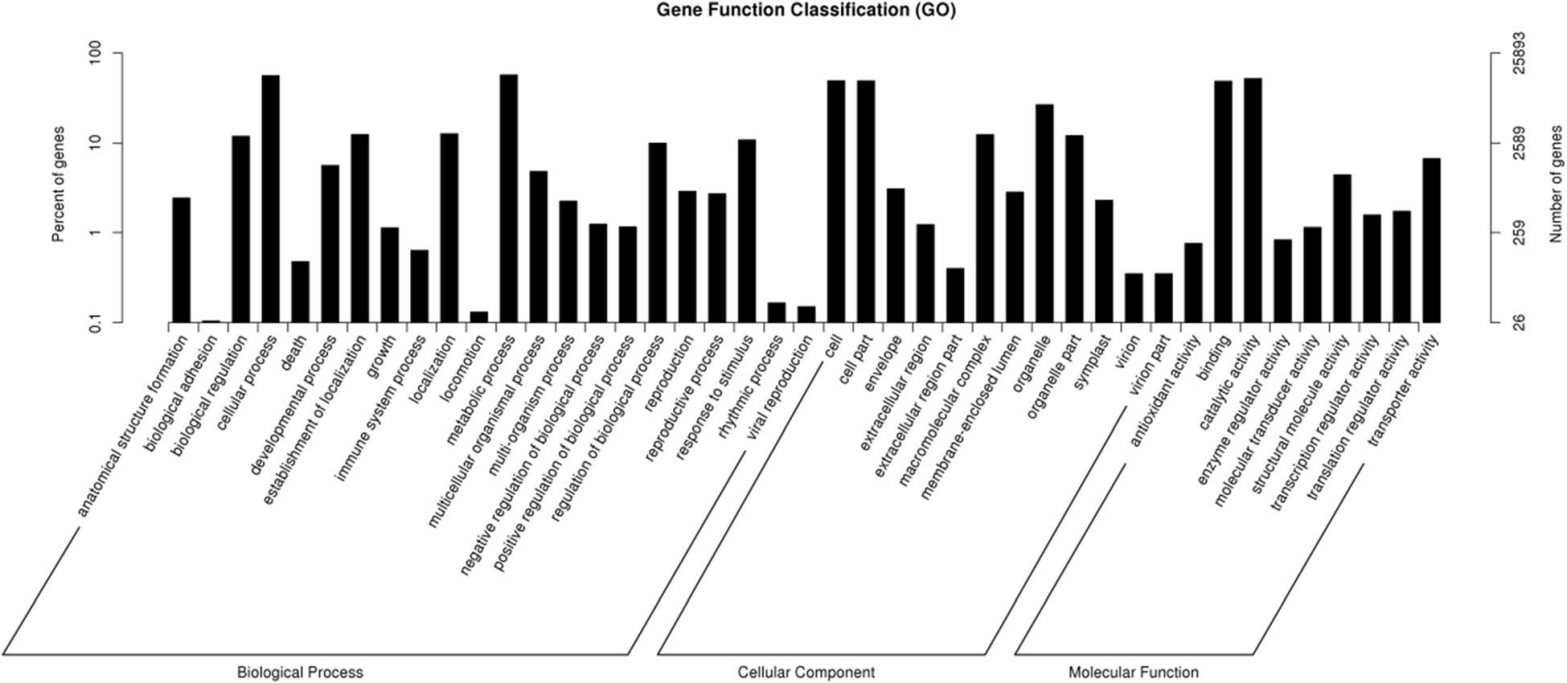
GO classification of the differentially expressed *Symplocos paniculata* unigenes (assigned number > 10) at four different fruit development stages. The left and the right side of the panel show the percentage of genes and the number of genes that are classified in the three terms including biological process, cellular component, and molecular function

To understand specific functions of the putative unigenes, BLAST search was conducted in the GO database. The GO database is a collection of the controlled vocabularies describing the biology of a gene product in any organism [26]. The 25,893 of the unigenes were assigned into three main GO functional categories (biological process, cellular component, molecular function) and 49 sub-categories (Fig. 3, Additional file 4: Table S2). The biological process category was assigned into 24 sub-categories. The most two abundant sub-categories were “metabolic process” and “cellular process”, which contained 14,754 unigenes (56.98% of the total) and 14,483 unigenes (55.93% of the total), respectively. The cellular component category was further classified into 14 sub-categories. The largest two sub-categories were “cell” and “cell part”, which contained 12,693 and 12,658 unigenes, respectively. The molecular function category had been mapped into 11 GO terms with the majority unigenes in “catalytic activity” (13,443 unigenes) and followed by “binding” (12,558 unigenes). These results suggested that a large number of metabolic activities occurred during the growth and development of *S. paniculata*.

**Fig. 4:**
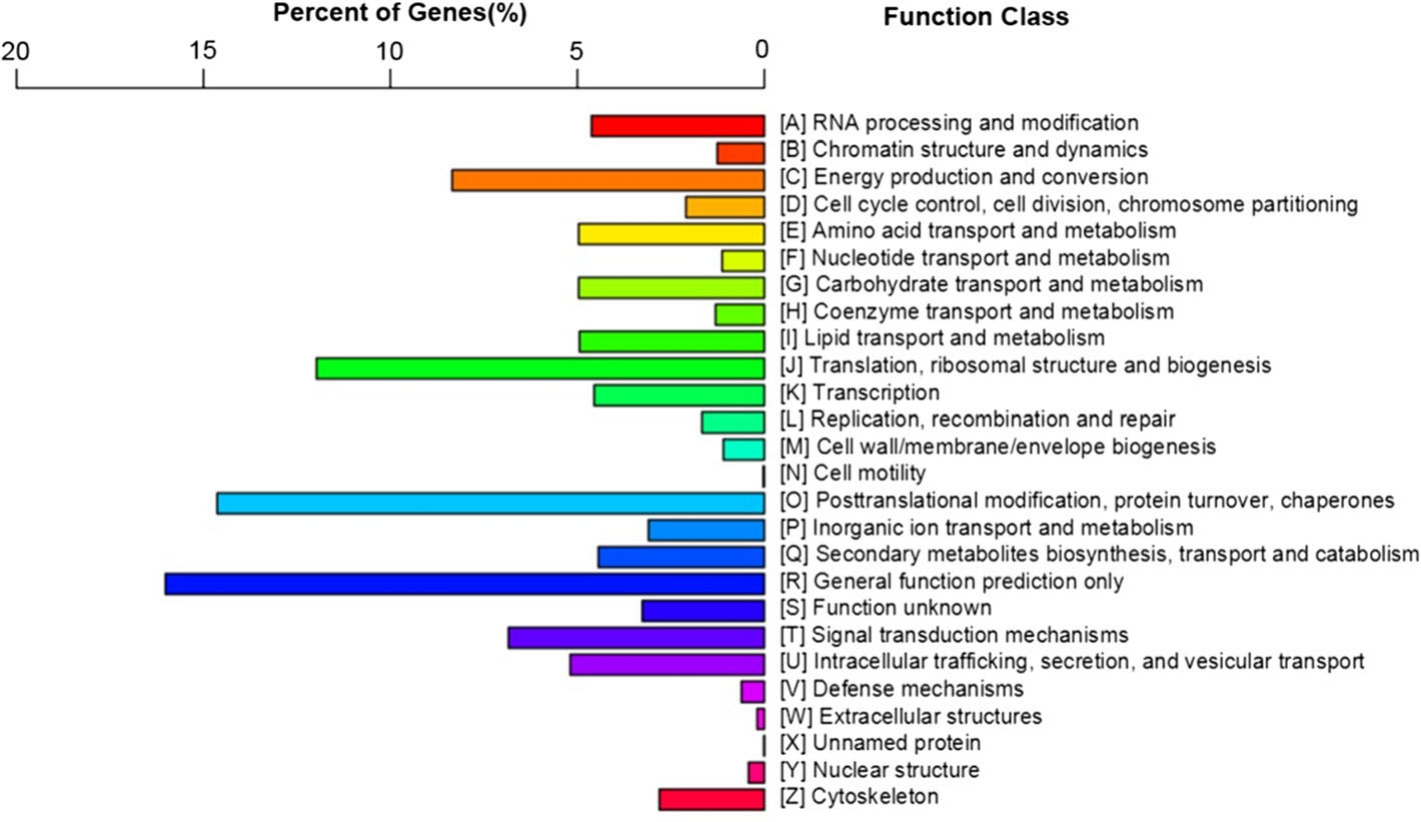
COG classifications of *Symplocos paniculata* unigenes

Meanwhile, COG analysis was used to determine the functions of the predicted unigenes [27]. The 26,831 unigenes were assigned into 26 function categories (Fig. 4, Additional file 5: Table S3). The largest group is “general function prediction only” (4,298, 16.02%). This indicated that a large number of unknown genes in *S. paniculata* that were deposited in the public database have a great exploration potential. The second largest group is “posttranslational modification, protein turnover, chaperones” (3,926, 14.58%), followed by “translation, ribosomal structure and biogenesis” (3,214, 11.98%), “energy production and conversion” (2,240 8.35%) and “signal transduction mechanisms” (1,836, 6.84%). However, the group of “cell motility” only contained 8 unigenes (0.02%). Of the unigenes, both the group of “carbohydrate transport and metabolism” and the group of “amino acid transport and metabolism” contained 1,332 (4.96%) unigenes, whereas the group of “lipid transport and metabolism” had 1,326 (4.94%) unigenes.

**Fig. 5:**
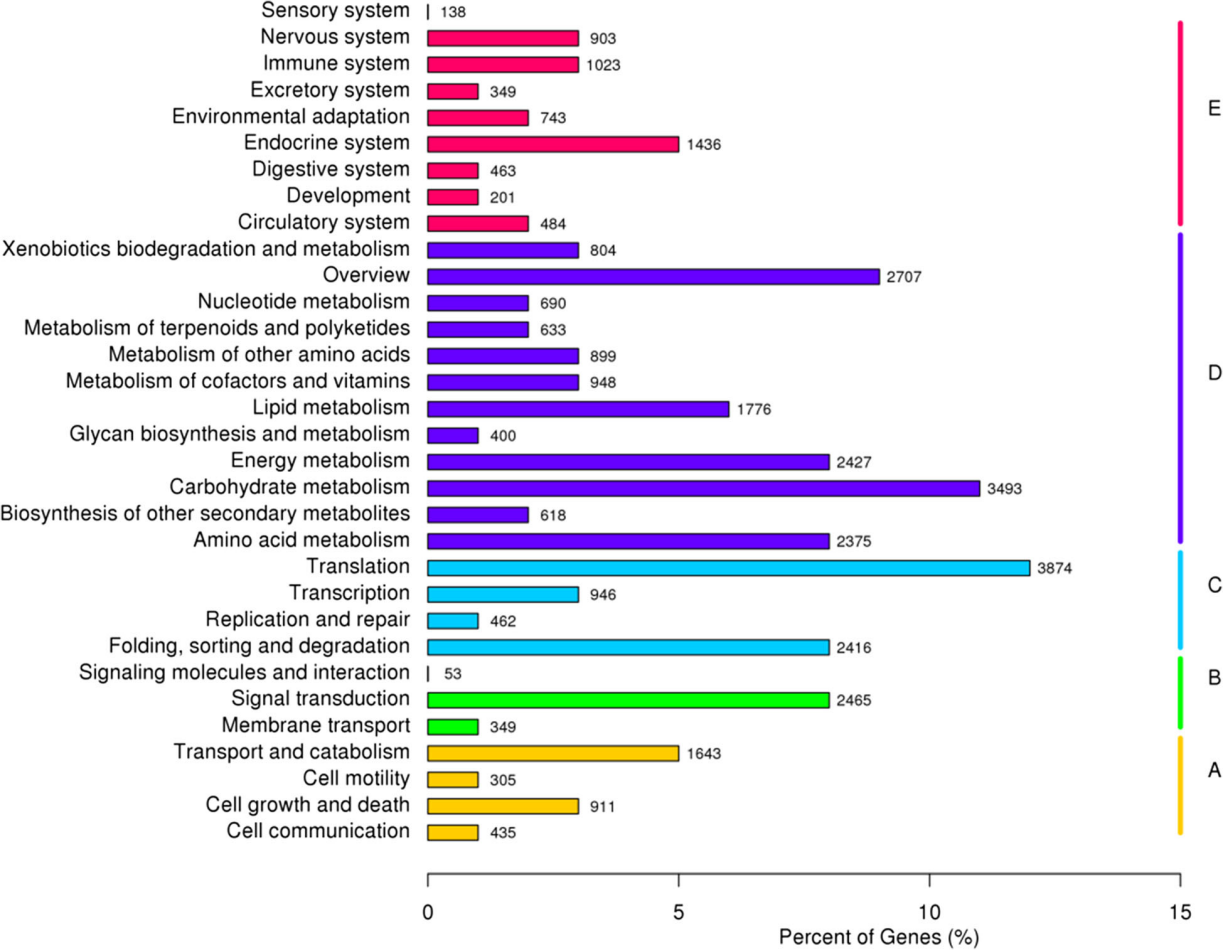
Functional classification and pathway assignment of unigenes of *Symplocos paniculata* by KEGG. The results are summarized in five main categories: **a** Cellular Process; **b** Environmental Information Process; **c** Genetic Information Process; **d** Metabolism; **e** Organismal Systems

The previously annotated sequences for unigenes involved in KEGG classifications have been searched to evaluate the completeness of transcriptome libraries and the effectiveness of the annotation processes [28]. The 31,407 unigenes were annotated into five KEGG categories (A: cellular process, B: environmental information process, C: genetic information process, D: metabolism, E: organismal systems), 32 sub-categories, and 284 pathways (Fig. 5, Additional file 6: Table S4). Among the five main categories, “metabolism” had the largest number of unigenes (15,395), followed by “genetic information processing” (7,698 unigenes), “organismal systems” (5,740 unigenes), “cellular processes” (3,294 unigenes) and “environmental information processing represented” (2,867 unigenes). Among the 32 sub-categories, “translation” was the maximum group with 3,874 unigenes, followed by “carbohydrate metabolism” (3,493 unigenes), and the smallest group containing only 53 unigenes was “signaling molecules and interaction”. Of the 284 pathways, approximately 1,776 unigenes were mapped to 16 lipid metabolic canonical pathways. Among them, “fatty acid metabolism” had the highest unigenes number (249 unigenes), followed by “glycerolipid metabolism” (212 unigenes), “fatty acid biosynthesis” (148 unigenes), “steroid biosynthesis” (145 unigenes), “glycerophospholipid metabolism” (142 unigenes), and “sphingolipid metabolism” (139 unigenes) (Additional file 7: Figure S3). “Primary bile acid biosynthesis” contained only 24 unigenes. In addition, other pathways related to lipid metabolism were “fatty acid elongation” (69 unigenes), “biosynthesis of unsaturated fatty acids” (112 unigenes), and “alpha-linolenic acid metabolism” (126 unigenes).

### Unigenes related to fatty acid biosynthesis

**Fig. 6:**
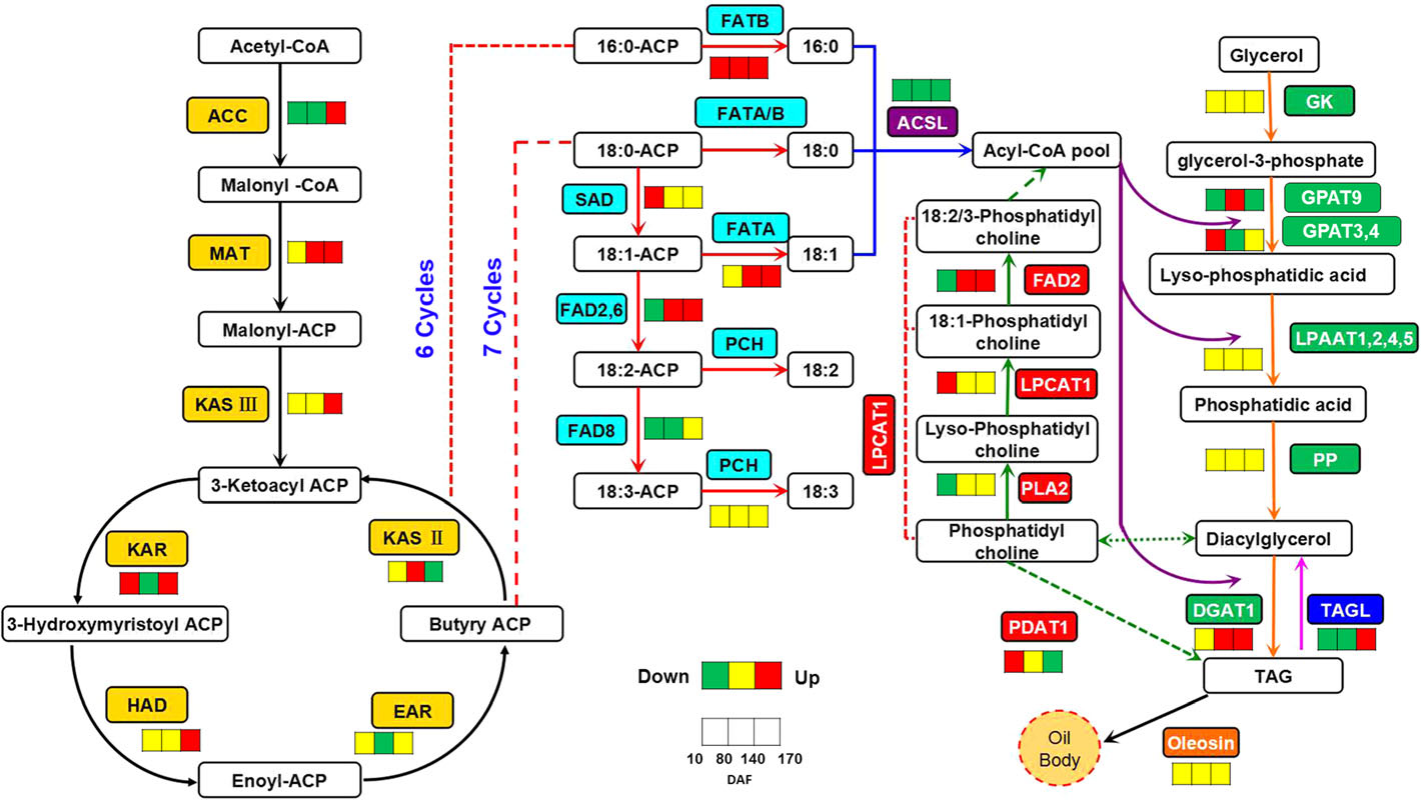
The schematic diagram of the pathway and temporal expressional patterns of lipid metabolism. The enzymes identified from functional unigenes annotation were used to produce the schematic diagram. The icons close to each enzyme show the results of DESeq analysis [10–80 DAF (*left*), 80–140 DAF (*middle*), and 140–170 DAF (*right*); *red*: up-regulation; *yellow*: no significant difference expression; *green*: down-regulation]. The identified key enzymes involved in lipid metabolism include acetyl-CoA carboxylase carboxyl transferase, (ACCase, EC:6.4.1.2); Malonyl-CoA-ACP transacylase, (MAT, EC:2.3.1.39); 3-Ketoacyl ACP synthase II, (KASII, EC:2.3.1.179); 3-Ketoacyl ACP synthase III, (KAS III, EC: 2.3.1.180); 3-Ketoacyl ACP reductase, (KAR, EC:1.1.1.100); 3R-hydroxymyristoyl ACP dehydrase, (HAD, EC:4.2.1.-); enoyl-ACPreductase I, (EAR, EC:1.3.1.9); fatty acyl-ACP thioesterase A, (FATA, EC:3.1.2.14); fatty acyl-ACP thioesterase B, (FATB, EC:3.1.2.14 3.1.2.21); acyl-ACP desaturase, (SAD, EC:1.14.19.1); palmitoyl-CoA hydrolase, (PCH, EC:3.1.2.2); long-chain acyl-CoA synthetase, (ACSL, EC:6.2.1.3); D12(v6)-Desaturase, (FAD2/6, EC:1.14.19.6); D15(v3)-Desaturase, (FAD8, EC:1.14.19.35); glycerol kinase, (GK, EC:2.7.1.30); glycerol-3-phosphate acyltransferase, (ATS1/GPAT, EC:2.3.1.15); lysophosphatidyl acyltransferase, (LPAAT, EC:2.3.1.51); phosphatidate phosphatase, (PP, EC:3.1.3.4); diacylglycerol O-acyltransferase 1, (DGAT1, EC:2.3.1.20); phospholipid: diacylglycerol acyltransferase 1, (PDAT1, EC:2.3.1.158); lysophosphatidylcholine acyltransferase, (LPCAT, EC:2.3.1.23 2.3.1.67) and phospholipase A2, (PLA2, EC:3.1.1.4). Lipid substrates are abbreviated: 16:0, palmitic acid; 18:0, stearic acid; 18:1, oleic acid; 18:2, linoleic acid and 18:3, linolenic acid

According to the KEGG and KOBAS pathway assignment and functional annotation of the unigenes, the key enzymes involved in lipid metabolism pathways had been found and presented in Table 3 and Additional file 8: Table S5. A sketch map of the lipid metabolism processes of *S. paniculata* fruit was produced on the basis of these identified enzymes, including fatty acid biosynthesis, fatty acid metabolism, glycerolipid metabolism, and glyceropholipid metabolism pathways (Fig. 6).

**Table 3:** Key enzymes related to fatty acid biosynthesis and metabolism identified by annotation of the *Symplocos paniculata* unigenes

Symbol	Enzymes	EC number	Unigene Number
Fatty acid biosynthesis			
ACC	acetyl-CoA carboxylase	[EC:6.4.1.2]	28
accA	acetyl-CoA carboxylase carboxyl transferase	[EC:6.4.1.2]	2
accB, bccP	acetyl-CoA carboxylase biotin carboxyl carrier protein	-	2
accC	acetyl-CoA carboxylase, biotin carboxylase	[EC:6.4.1.2 6.3.4.14]	4
MAT	malonyl-CoA-ACP transacylase	[EC:2.3.1.39]	1
KAR	3-oxoacyl-[ACP] reductase	[EC:1.1.1.100]	28
HAD	3-hydroxyacyl-[ACP] dehydratase	[EC:4.2.1.59]	3
EAR	enoyl-[ACP] reductase I	[EC:1.3.1.9 1.3.1.10]	4
KASII	3-oxoacyl-[ACP] synthase II	[EC:2.3.1.179]	15
KASIII	3-oxoacyl-[ACP] synthase III	[EC:2.3.1.180]	2
FATA	fatty acyl-ACP thioesterase A	[EC:3.1.2.14]	1
FATB	fatty acyl-ACP thioesterase B	[EC:3.1.2.14 3.1.2.21]	5
Fatty acid elongation			
KCS	3-ketoacyl-CoA synthase	[EC:2.3.1.199]	18
PCH	palmitoyl-CoA hydrolase	[EC:3.1.2.2]	4
Fatty acid desaturation			
FAD2	omega-6 fatty acid desaturase (delta-12 desaturase)	[EC: 1.14.19.6]	36
FAD6	omega-6 fatty acid desaturase (delta-12 desaturase)	[EC: 1.14.19.6]	3
FAD8	omega-3 fatty acid desaturase (delta-15 desaturase)	[EC: 1.14.19.35]	5
SAD	stearoyl-ACP desaturase	[EC:1.14.19.1]	52
AAD	acyl-[ACP] desaturase	[EC:1.14.19.2]	8
Fatty acid metabolism			
MFP2	enoyl-CoA hydratase/3-hydroxyacyl-CoA dehydrogenase	[EC:4.2.1.17 1.1.1.35 1.1.1.211]	5
ACOX	acyl-CoA oxidase	[EC:1.3.3.6]	29
ACSL	long chain acyl-CoA synthetase	[EC:6.2.1.3]	70
ACAA	acetyl-CoA acyltransferase	[EC:2.3.1.16]	22
ACAA1	acetyl-CoA acyltransferase 1	[EC:2.3.1.16]	4
ATOB	acetyl-CoA C-acetyltransferase	[EC:2.3.1.9]	19

The vital enzymes in the pathway of fatty acid biosynthesis were identified. Firstly, 28 unigenes were identified to encode multi-subunit acetyl-CoA carboxylase (ACC, EC: 6.4.1.2). ACC is a rate-limiting enzyme to catalyze acetyl-CoA to form malonyl-CoA in the fatty acid biosynthesis pathway. Secondly, only one unigene was found for coding the enzyme of malonyl-CoA ACP transacylase (MAT, EC: 2.3.1.39). MAT catalyzes the malonyl-CoA to the malonyl-ACP, which is the primary substrate for a subsequent cycle of condensation reactions. In condensation reaction cycle, two carbon units of acyl chain were added to malonyl-ACP with the help of several key enzymes for fatty acid synthesis. These enzymes were identified including 3-ketoacyl-ACP synthase III (KAS III, EC: 2.3.1.180), 3-ketoacyl ACP reductase (KAR, EC: 1.1.1.100), 3-Hydroxyacyl-ACP dehydratase (HAD, EC: 4.2.1.59), enoyl-ACP reductase I (EAR, EC: 1.3.1.9), and 3-ketoactyl-ACP synthase II (KAS II, EC: 2.3.1.179). This condensation reaction cycle was repeated six or seven times to add a total of 12 or 14 carbon units. Through these processes, the acetyl-CoA consecutively turns into palmitic acid-ACP (16:0-ACP) or generates stearic acid-ACP (18:0-ACP). Along the pathway of fatty acid elongation, one unigene was found to encode fatty acyl-ACP thioesterase A (FATA, EC: 3.1.2.14) that release 18:0/1-ACP to 18:0/1. Five unigenes to encode fatty acyl-ACP thioesterase B (FATB, EC: 3.1.2.14 3.1.2.21) that prefer to remove ACP from 16:0-ACP to produce 16:0, and four unigenes to encode PCH (EC: 3.1.2.2) that release 18:2/3-ACP to 18:2/3. Meanwhile, in the pathway of fatty acid desaturation, 52 unigenes were identified to encode acyl-ACP desaturase (SAD, EC: 1.14.19.1) that desaturates18:0-ACP to 18:1-ACP. A total of 39 unigenes were introduced to encode D12 (v6)-Desaturase (36 for FAD2 and 3 for FAD6, EC: 1.14.19.6) that can desaturate 18:1-ACP to 18:2-ACP. Five unigenes were encoded D15 (v3)-Desaturase (FAD8, EC: 1.14.19.35) that further desaturates 18:2-ACP to form 18:3-ACP. Furthermore, 70 unigenes were encoded long chain acyl-CoA synthetase (ACSL, EC: 6.2.1.3) was involved in the fatty acid metabolism pathway (Fig. 6; Table 3), which was responsible for conversion of acyl-CoAs pool from free fatty acids. Acetyl-CoA generated provides an intermediate for the TAG synthesis process by fatty acid catabolism.

In our study, no unigene was found to encode the 3-ketoactyl-ACP synthase I (KAS I), which shares a partial of functions with KAS II in fatty acid elongation pathways. KAS could catalyze to extend the carbon chain from C2 to C14, but is far less effective for 16:0-ACP and almost inactive for 18:0-ACP [37]. Oleic acid (C18:1) is a dominating component of the fruit oil of *S. paniculata*. Our observations indicate that KAS I is not an essential enzyme in *S. paniculata*, although it is very common in eukaryotes and bacteria [38]. Similar results were seen in other woody oil plants [39].

### Unigenes related to catabolism pathways for TAGs

Acylglycerols act as an energy reserve in many organisms and are the major components of seed oil [40]. TAG is the most common acylglycerol in seed oil. Free fatty acids serve as the primary substrate for TAG biosynthesis. All putative enzymes within the TAG biosynthesis pathway based on the KEGG pathway assignment were listed (Table 4 and Additional file 6: Table S4), and the TAG pathway was shown in Fig. 6. A total of 14 unigenes were found to code glycerol kinase (GK, EC: 2.7.1.30) that catalyzed glycerol to produce the glycerol-3-phosphate. The glycerol-3-phosphate was subsequently catalyzed to form lysophosphatidic acid with the help of 24-unigenes-coded glycerol-3-phosphate acyltransferase (GPAT, EC: 2.3.1.15) (two for GPAT1, one for GPAT3, two for GPAT4, two for GPAT7, one for GPAT8, and 16 for GPAT9). Four kinds of acylglycero-3-phosphate acyltransferase (LPAAT, EC: 2.3.1.51) had been identified to LPAAT1, LPAAT2, LPAAT4, and LPAAT5 with five, two, three, and one encoded unigenes respectively. They played a critical role in acylating the lyso-phosphatidic acid at the position sn-2 to synthesize phosphatidic acid (PA) in the plastid, the endoplasmic reticulum, and the mitochondria of cells [41]. Six unigenes were found to encode phosphatidate phosphatase (PP, EC: 3.1.3.4) that can remove the phosphate group of the PA to generate diacylglycerols (DAG). At the last step of TAG biosynthesis, six-unigene-encoded diacylglycerol acyltransferase (DGAT, EC: 2.3.1.20) transferred an acyl group from acetyl-CoA to sn-3 of DAG to form TAG. TAGs stored in spherical compartments in oil body form serve as energy sources for seed germination and seedling growth [42]. Oil bodies are surmised to arise from the endoplasmic reticulum (ER) [43, 44] and are surrounded by a phospholipid monolayer and abundant amphipathic proteins such as oleosin, caleosin, and steroleosin [45–47]. In our transcriptome libraries, six transcripts were found to code oleosin, two unigenes for caleosin, and one for steroleosin. Alternatively, TAG can transform from a phosphatidylcholine (PC) with the aid of the phospholipid: diacylglycerol acyltransferase (PDAT, EC: 2.3.1.158) (seven unigenes), which uses phosphatidylcholine as the acyl donor to transfer an acyl group to the sn-3 position of DAG to produce TAG [48]. It is generally accepted that phosphatidylcholine (PC) also can be used for polyunsaturated fatty acids synthesis, one unigene was identified to encode phospholipase A2 (PLA2, EC: 3.1.1.4) that catalyzes PC to generate lysophosphatidylcholine. And four unigenes encodes lysophosphatidylcholine acyltransferase (LPCAT1, EC: 2.3.1.23) that esterifies oleic acid (C18:1) to form C18:1-PC, and then C18:1-PC was desaturated by FAD2 to generate C18:2/3-PC.

**Table 4:** Key enzymes related to TAG biosynthesis and metabolism identified by annotation of the *Symplocos paniculata* unigenes

Symbol	Enzymes	EC number	Unigene number
TAG biosynthesis			
GK	glycerol kinase	[EC:2.7.1.30]	14
GPAT1	glycerol-3-phosphate O-acyltransferase1	[EC:2.3.1.15]	2
GPAT3	glycerol-3-phosphate O-acyltransferase 3	[EC:2.3.1.15]	1
GPAT4	glycerol-3-phosphate O-acyltransferase 4	[EC:2.3.1.15]	2
GPAT7	glycerol-3-phosphate O-acyltransferase 7	[EC:2.3.1.15]	2
GPAT8	glycerol-3-phosphate O-acyltransferase 8	[EC:2.3.1.15]	1
GPAT9	glycerol-3-phosphate acyltransferase 9	[EC:2.3.1.15]	16
LPAAT1	lysophospholipid acyltransferase	[EC:2.3.1.51]	5
LPAAT2	lysophosphatidate acyltransferase	[EC:2.3.1.51]	2
LPAAT4	lysophosphatidylinositol acyltransferase	[EC:2.3.1.51]	3
LPAAT5	1-acyl-sn-glycerol-3-phosphate acyltransferase	[EC:2.3.1.51]	1
PP	phosphatidate phosphatase	[EC:3.1.3.4]	6
DGAT1	diacylglycerol O-acyltransferase 1	[EC:2.3.1.20]	6
PDAT1	phospholipid:diacylglycerol acyltransferase	[EC:2.3.1.158]	7
PLA2	phospholipase A2	[EC:3.1.1.4]	1
LPCAT1	lysophosphatidylcholine acyltransferase	[EC:2.3.1.23]	4
plcC	phospholipase C	[EC:3.1.4.3]	8
TAZ	monolysocardiolipin acyltransferase	[EC: 2.3.1.-]	8
OLE	oleosin	-	6
CLO1	caleosin	-	2
SLO1	steroleosin	-	1
TAG metabolism			
TAGL	triacylglycerol lipase	[EC:3.1.1.3]	29
MAGL	monoacylglycerol Lipase	[EC:3.1.1.23]	1
EPT1	ethanolaminephosphotransferase	[EC:2.7.8.1]	6

In TAG metabolism, 26-unigene-encoded triacylglycerol lipases (TAGL, EC: 3.1.1.3) release TAG to DAG. It is worth mentioning that the DGAT, PDAT, and TAGL are vital enzymes in the TAG pathway that determine cellular oil content and quality. Results in this study establish a nearly complete gene bank associated with oil accumulation of *S.paniculata*. Expressed sequence tags (ESTs) revealed could be considered candidate genes for future genetic cloning and modification.

### Gene expression profiles of oil accumulation

**Fig. 7:**
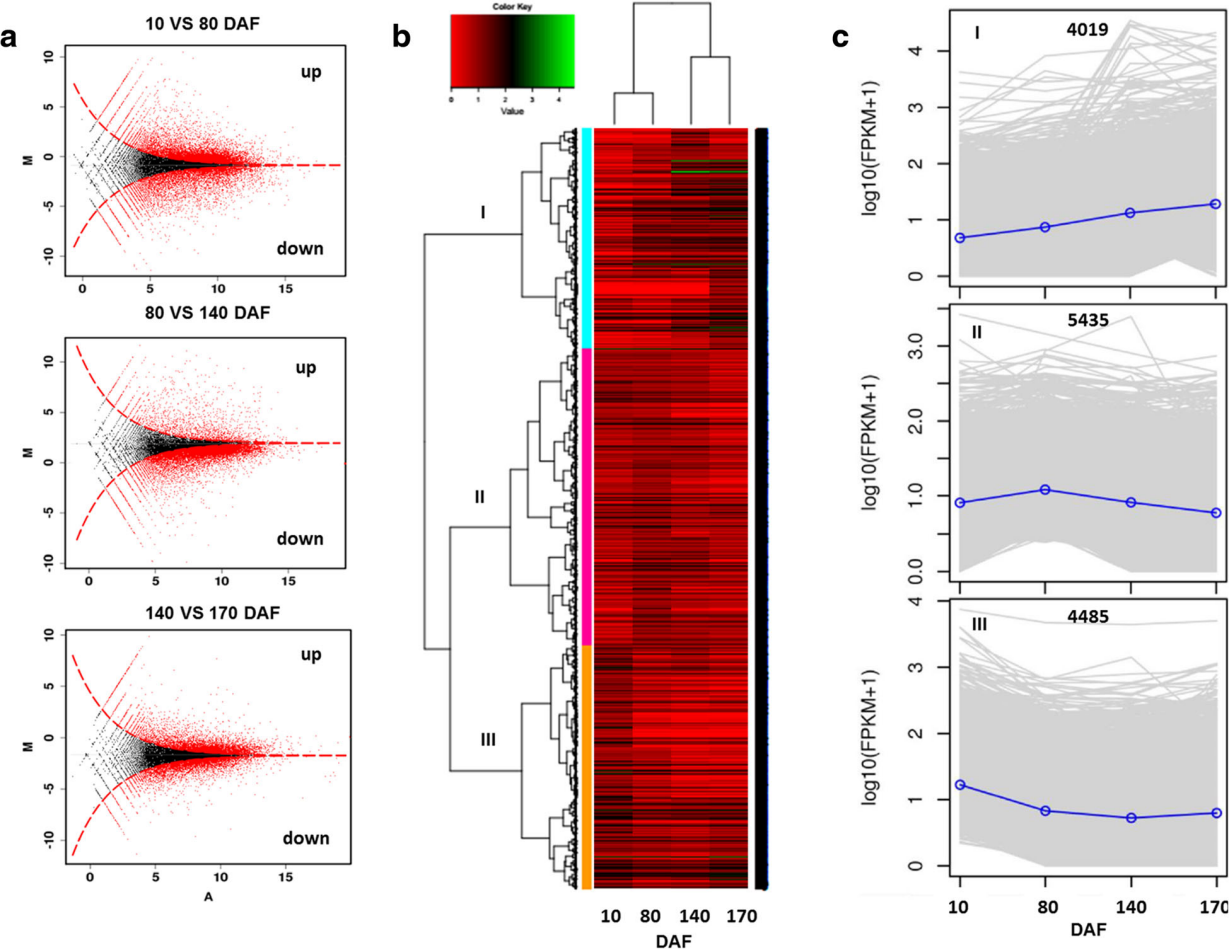
Cluster analysis of the differentially expressed genes in *Symplocos paniculata*. **a** Comparative profiles of differentially expressed unigenes level during the four oil accumulation period, from top to bottom: 10 vs 80 DAF, 80 vs 140 DAF, and 140 vs 170 DAF, A: Log_2_ (*read counts for each gene*), M: Log_2_(RPKM ratio); **b** Dendrogram of hierarchical cluster analysis of the differentially expressed unigenes (DEGs), The *green* highlights genes being highly expressed. The *red* highlights genes being low expressed. The color scale indicates unigenes expression values; **c** The three cluster group of different expressional patterns

In order to fully understand the differentially expression patterns of the specific genes associated with fruit development and oil accumulation. By comparing RPKM value of unigenes between the different fruit oil accumulation phases of *S. paniculata* (Additional file 9: Figure S4 and Additional file 10: Table S6), a total of 13,939 unigenes increased or decreased over 3-folds in RPKM value and were differentially expressed in our experiment (Fig. 7a). A hierarchical cluster analysis was conducted based on the RPKM value of these unigenes (Fig. 7b). All differentially expressed unigenes were clustered into three groups. Unigenes in a single cluster have identical or similar expression patterns during fruit oil accumulation stages. Group I consisted of 4,019 DEGs that all represented up-regulated pattern. The mean log_2_RPKM value of the DEGs continues to increase with the fruit development. Group II contained 5,435 DEGs that up-regulated from 10 to 80 DAF and down-regulated thereafter. Group III included 4,485 DEGs that were down-regulated (Fig. 7c). Cluster results suggest that a relatively clear and coordinated expression pattern occurs during the fruit development period of *S. paniculata*.

**Fig. 8:**
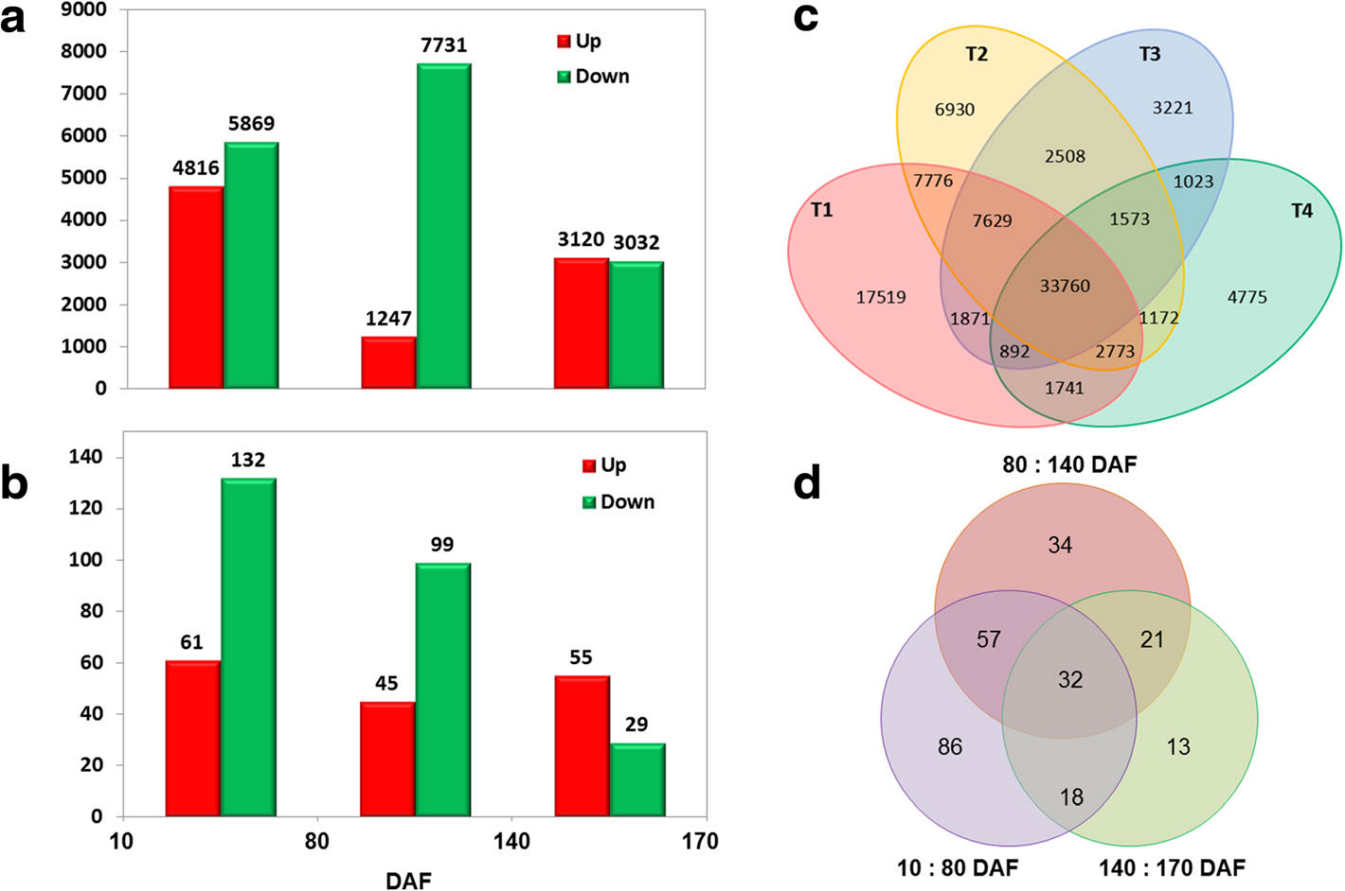
Differentially expressed profiles of unigene among the different fruit developmental stages of *Symplocos paniculata*. **a** Distribution of up and down regulated unigenes in different development stages, **b** Distribution of up and down regulated unigenes involved in lipid metabolism in different development stages, **c** Distribution of unigenes in different fruit development stages, **d** Distribution of differential expression unigenes involved in lipid metabolism

The up and down regulated unigenes at different developmental stages were integrated (Fig. 8a) and mapped into the GO (Additional file 11: Table S7) and KEGG public databases (Additional file 12: Table S8). The distribution of up-regulated and down- regulated unigenes involved in lipid metabolism pathway was described in Fig. 8b. A total of 193 unigenes were differentially expressed genes between 10 and 80 DAF with more down-regulated unigenes (132) than up-regulated ones (61). Between 80 and 140 DAF, 144 unigenes were greatly expressed with more down-regulated unigenes (99) than up-regulated ones (45). However, 84 unigenes differentially expressed between 140 and 170 DAF, and the number of up-regulated ungenes (55) was more than that of down-regulated unigenes (29). The results of a Venn diagram analysis showed that 33,760 unigenes were expressed in all four samples (Fig. 8c). There were 17,519, 6,930, 3,221, and 4,775 unigenes expressed in 10, 80, 140, and 170 DAF, respectively. A comparative analysis of differentially expressed unigenes related to lipid metabolism in the four developmental stages identified 86, 34, and 13 differentially expressed unigenes during the period of 10–80, 80–140, and 140–170 DAF (Fig. 8d). The expression pattern of differential regulatory unigenes might play an essential role in modulating the oil accumulation of *S. paniculata*.

The up and down regulated unigenes involved in lipid metabolism were annotated (Fig. 6 and Additional file 8: Table S5). A strong temporal pattern in transcriptional profiles throughout oil synthesis was observed. During the period of 10 to 80 DAF (10 DAF as a control), enzymes including KAR, FATB, SAD, MFP2, LPCAT1,GPAT3,4 and PDAT1 were up-regulated whereas the enzymes of ACC, FAD2, FAD8, PLA2, and GPAT9 involved in lipid metabolism were mostly down-regulated. These results indicate that genes related to fatty acids or TAG metabolisms were relatively inactive which resulted in the slow oil accumulation. In contrast, carbohydrate metabolism is more active at the early fruit development phase. Carbohydrate metabolism and lipid metabolism share the same substrate of acetyl-CoA, which is an important precursor for *de novo* fatty acid biosynthesis and elongation, tricarboxylic acid cycle (TCA), and other phytochemical biosynthesis in plant cells [49].

During the period of 80 to 140 DAF, the up-regulated unigenes associated with FAs and TAG biosynthesis pathways, such as MAT, EAR, KAS II, FATA, FATB, FAD2, GPAT9, and DGAT1 were highly expressed at this stage of fruit development. These also correspond with the increase of oil content in fruit (Fig. 6, Additional file 1: Figure S1). These candidate unigenes associated with oil accumulation pathways showed great potential for further genetic modification of oil content improvement. During the period of 140 to 170 DAF, the up-regulated unigenes (KASIII, KAR, FATA, FATB, FAD2, ACAA, DGAT1, and TAGL) were notably linked to fatty acid elongation and fatty acid desaturation pathways. This coincided with our investigation on dynamic patterns of oil content and fatty acid composition. For example, unsaturated fatty acid increased sharply with stable oil content between 140 to 170 DAF. FAD2 and FAD8 played essential roles in the conversion of saturated fatty acid to unsaturated fatty acid.

It is well known that acyl-CoAs is transported from the cytosol to ER for glycerolipid synthesis through diffusion via soluble carriers or more efficient inter-membrane transporters [10]. ACC is a key enzyme in the *de novo* fatty acid biosynthesis pathway. Overexpression of ACC alters the fatty acid composition of seed oil and increases the fatty acid content, which leads to an increased oleic acid content [50, 51]. In our study, an extremely low expression of ACC was observed. The expression of ACC was down-regulated at 80 and 140 DAF and up-regulated at 170 DAF (Fig. 6). Similar results were seen in Siberian apricot (*Prunus sibirica*) [15] and tung oil tree (*Vernicia fordii*) [52]. A low expression of ACC genes at 80 and 140 DAF might be related to the remarkable accumulation of fatty acids in this development stage (oleic acid, C18:1 is one major component of *S. paniculata*) (Additional file 2: Figure S2). This agrees with previous reports that adding oleic acid (C18:1) to the cell suspension culture of canola (*B. napus*) could inhibit plastidial ACC activity and cause a reduction of fatty acid synthesis [53]. Modifying the ACC to improve lipid production was attended, but not effective [54]. Therefore, further research should be conducted to better understand ACC transcriptional regulatory mechanisms that contribute to the oil synthesis.

FATA/B thioesterases make a remarkable contribution for free fatty acids synthesis that releases the fatty acids from the acyl carrier protein (ACP). FATB is more effective for removing ACP from 16:0-ACP to produce 16:0, while FATA prefer to hydrolyze 18:0-ACP or 18:1-ACP to release ACP to generate stearic acid (C18:0) and oleic acid (C18:1) [10]. In this study, FATA genes showed a stable expression between 10 to 80 DAF and sharply up-regulated between 80 to 140 DAF (6 fold) and 140 to 170 DAF. This coincides with the rapid increase in oleic acid (C18:1) (Fig. 6; Additional file 2: Figure S2). However, the FATB genes notably up-regulated throughout fruit development that resulted in a higher proportion formation of palmitic acid (C16:0) over stearic acid (C18:0) and oleic acid (C18:1) during the period of 10 to 80 DAF (Fig. 6; Additional file 2: Figure S2). Gene manipulation of FATA/B could be done to adjust the relative proportion of fatty acids.

Two types of enzymes participate in fatty acid desaturation in *S. paniculata*. One type includes AAD and SAD that catalyze saturated fatty acid in plastids (C18:0-ACP to C18:1-ACP) to form monounsaturated fatty acids. The other type is located on the membranes of the endoplasmic reticulum and chloroplast and introduces double-unsaturated bonds at specific positions. For example, FAD2 and FAD6 catalyze unsaturated C18:1-ACP to C18:2-ACP. FAD8 further desaturates C18:2-ACP to form C18:3-ACP [55]. In our study, SAD genes were up-regulated over five folds during the period of 10 to 80 DAF when a concomitant sharp rising of oleic acid (C18:1) occurred. FAD2 genes were down-regulated during the period of 10 to 80 DAF, but dramatically up-regulated over six folds during the period of 80 to 140 and 140 to 170 DAF. This pattern corresponds to the trend of linoleic acid (C18:2). FAD8 genes were down regulated throughout fruit development with a stable expression of AAD and FAD6 (Fig. 6). Fatty acid composition of oil plants were genetically modified using the hairpin RNA-mediated gene silencing technique to down-regulate the expression of the key fatty acid desaturase genes in seeds [56]. In comparison with untransformed plants (control), the inhibition of the expression of SAD genes resulted in a 38% increase of stearic acid in both canola [56] and cotton (*Gossypium hirsutum*) [57] seed oils. Oleic acid (C18:1) content was also increased through silencing FAD2 by 26 28 76 and 62%, respectively, in canola and mustard greens (*B. juncea*) [58], soybean (*Glycine max*) [59], and cotton seeds [57]. In addition, palmitic acid decreased after genetic modification [56]. It is important to note that the relative proportion of fatty acids is the critical factor that impacts bio-diesel production and edible oil quality. The oil rich in monounsaturated fatty acids instead of saturated fatty acid and polyunsaturated fatty acid (PUFA) is ideal for bio-diesel production. High content of unsaturated fatty acids would contribute to the instability of bio-diesel fuel [60]. However, unsaturated fatty acids in edible oil, especially the oleic acid, play a significant role in human health [61]. *S. paniculata* oil has high percentages of oleic and linoleic acids. SAD, FAD2, and FAD6 are potential targets for modifying oil composition to meet the needs of either edible oil production or bioenergy applications.

PDAT1 and DGAT1 were two confirmed genes that are essential for TAG biosynthesis. In previous studies, silencing of PDAT1 or DGAT1 would result in 70 to 80% decreases in oil content [62]. In our study, DGAT1genes were up-regulated during the period of 80 to 170 DAF, whereas PDAT1 up-regulated expression between 10 and 80 DAF (Fig. 6). These results suggest that DGAT1 might be the major enzyme in the last step of TAG biosynthesis in *S. paniculata*. Moreover, ectopic expression of DGAT1 was confirmed to improve the oil content in seeds of *Arabidopsis* [63], maize (*Zea mays*) [64], and soybean [65]. Overexpression of DGAT1 would improve oil accumulation. Similarly, PDAT1 may provide a way to produce fatty acids from acetyl-CoA in TAG biosynthesis. In addition, TAGL is a key enzyme related to TAG metabolism that showed a down-regulation during the period of 10 to140 DAF and up- regulation between 140 and 170 DAF (Fig. 6). TAGL metabolized TAG to DAG and thus, lead to oil content reduction. These suggest the inhibition of TAGL genes would decrease TAG metabolism to increase lipid storage.

GPAT is a key enzyme that plays a critical role in the first step of biosynthesis of membrane phospholipids and storage TAG in nearly all plants [66]. GPAT catalyzes glycerol-3-phosphate to generate lysophosphatidic acid. LPAAT then catalyzes the subsequent acylation of lysophosphatidic acid at the sn-2 position to produce phosphatidic acid. Phosphatidic acid is a key intermediate in the biosynthesis of both membrane polar lipid and neutral storage lipid [67]. In previous studies on the GPAT family in *Arabidopsis*, GPAT1 and GPAT4-8 with sn-2 region specificity were non-essential for TAG synthesis, whereas GPAT9 was the ER-localized GPAT enzyme responsible for plant membrane lipid and oil biosynthesis [66]. In addition, the function of GPAT2-3 still remains unclear [68, 69]. Genetic modification of GPAT and LPAAT in the TAG assembly has been demonstrated to enhance seed oil content [70]. For example, overexpression of a plastidial safflower GPAT and an *Escherichia coli* GPAT in *Arabidopsis* can increase the seed oil content to 22 and 15%, respectively [71]. A notable increase of 8 and 48% in seed oil content were observed by overexpression of a mutant form of yeast LPAAT in *Arabidopsis* and canola [72]. Overexpression of the rapeseed microsomal LPAAT isozymic gene could result in a 13% increase in oil content of *Arabidopsis* seeds [73]. Results indicate that increasing the expression of LPAAT in seeds might lead to a greater flux of intermediates through the Kennedy pathway [74] and result in TAG accumulation. In our study, GPAT3, GPAT4, GPAT7, GPAT8, and GPAT9 showed different expression patterns at fruit development stages indicating that they function differently (Fig. 6, Additional file 6: Table S4). GPAT9 is up-regulated between 80 and 140 DAF that coincide with rapid oil increase (Fig. 6, Additional file 1: Figure S1). But the LPAAT genes family did not show any significant expression (Fig. 6, Additional file 6: Table S4). Therefore, improving GPAT9 production would contribute to the oil synthesis of *S. paniculata*.

### Experimental validation and analysis of key enzymes involved in lipid metabolism

**Fig. 9:**
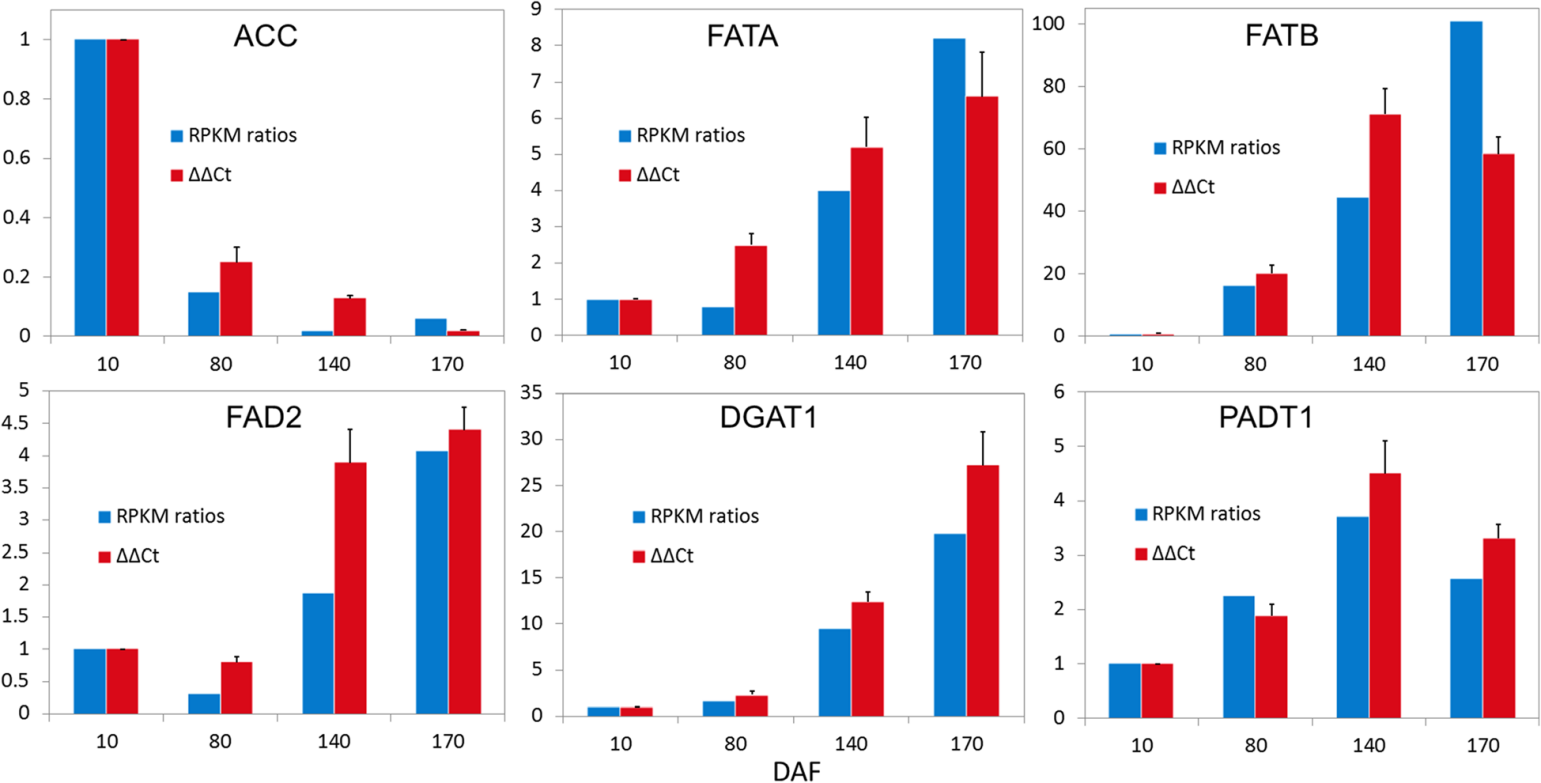
Quantitative RT-PCR validations of the six candidate lipid-related genes of *Symplocos paniculata*. The comparative RPKM ratio and ΔΔCt at 10 DAF are used as a control for normalization. Results represent the mean (±SE) of three biological replicates. Error bars represent the standard error of three biological replicates

The relative expression level and temporal transcription patterns of the key genes associated with oil accumulation were analyzed [15] to assess the accuracy of the sequencing and target of *S. paniculata* transcriptome. Six vital enzymes including ACC, FATA, FATB, FAD2, DGAT1, and PADT1 were selected to design primers for qRT-PCR validation (Additional file 13: Table S9). The ΔΔCt values of these selected genes were mostly consistent with sequencing results (Fig. 9). There were significant correlations between RPKM with ΔΔCt with correlation coefficient of 0.9879, 0.9250, 0.7558, 0.8801, 0.9996, and 0.9608 for ACC, FATA, FATB, FAD2, DGAT1, and PADT1, respectively (Additional file 13: Table S9). These results indicate that the unigenes assembly results were reliable and it is feasible to use the DESeq method to investigate subsequent differential expression analysis. The expression level of most of the selected genes was higher in the qRT-PCR validation experiment than in the sequencing analysis with the exception of ACC, FATA, and FATB at 170 DAF and PADT1 at 80 DAF. The extremely low expression level of ACC at 170 DAF made it difficult to detect. A different expression pattern of FATB was observed between qRT-PCR analysis and DESeq result. FAD2 genes exhibited higher expression in the qRT-PCR validation experiment at 140 DAF. Such inconsistency in the expression level of FATB and FAD2 genes could be due to primers’ specificity and qRT-PCR reaction conditions during the experimental validation analysis [75].

## Conclusions

In our study, the transcriptome of *S. paniculata* had been sequenced and annotated using Illumina RNA-seq technology. A total of 182,904 non-redundant unigenes were assembled and annotated in the NR, GO, COG, KEGG protein public database successfully. Based on further functional annotation of the KEGG protein public database, crucial enzymes controlling oil accumulation had been identified and an integrated pathway related to core lipid metabolism had been reconstructed. A total of 13,939 unigenes were determined having expressed difference using the DESeq method. The transcriptional regulation profiles along with temporally dynamic oil accumulation patterns were systematically analyzed. The key regulatory enzymes involved in lipid metabolism (ACC, KASII, KASIII, FATA, FATB, ACSL, SAD, FAD2, GPAT9, LPCAT1, DGAT1, and PDAT1) were determined and they play vital roles in the oil accumulation in *S. paniculata* fruit and fatty acid composition of the fruit oil. Moreover, the temporal expression levels of six key genes (ACC, FATB, FATA, FAD2, DGAT1, and PDAT1) were also validated using qRT-PCR. This was the first and most comprehensive investigation on the lipid genes annotation of *S. paniculata*. Results demonstrated that Illumina pyrosequencing possessed potential of rapidly capturing a large number of transcriptomes. The transcriptome sequences will massively enrich public databases and provide new insights into functional genes discoveries associated with lipid metabolic pathways in *S. paniculata*. Our results will serve as a foundation to explore transcriptional regulatory profiles of *S. paniculata* to elucidate the molecular regulatory mechanism and to accelerate the genetic modification to increase fruit oil content and quality. Results in this paper may also provide reference for other researches on woody oil plants.

## Additional files

Additional file 1: Figure S1. Dynamic change of fruit oil content during the fruit development. (PDF 33 kb)
Additional file 2: Figure S2. Dynamic change of the main fatty acids in fruit oil during the fruit development. (PDF 59 kb)
Additional file 3: Table S1. The distribution of sequencing reads in each library. (XLSX 9 kb)
Additional file 4: Table S2. Distribution of unigenes assigned into GO public database. (XLS 31 kb)
Additional file 5: Table S3. Distribution of unigenes assigned into COG public database. (XLS 21 kb)
Additional file 6: Table S4. Distribution of unigenes assigned into KEGG public database. (XLSX 22 kb)
Additional file 7: Figure S3. Distribution of unigenes classified to 16 lipid metabolism pathways. (PDF 384 kb)
Additional file 8: Table S5. Distribution and expression patterns of key enzymes involved in lipid metabolism. (XLSX 16 kb)
Additional file 9: Figure S4. The RPKM distribution of unigenes. (PDF 72 kb)
Additional file 10: Table S6. FPKM comparison of up and down regulated unigenes by DEGs. (XLS 5071 kb)
Additional file 11: Table S7. GO annotation of up and down regulated unigenes by DEGs. (XLS 54 kb)
Additional file 12: Table S8. KEGG annotation of up and down regulated unigenes by DEGs. (XLS 1411 kb)
Additional file 13: Table S9. The designed primers of the key enzymes involved in lipid metabolism for qRT-PCR. (XLS 26 kb)

## Abbreviations

18:0: Stearic acid
18:1: Oleic acid
18:2: Linoleic acid
18:3: Linolenic acid
ACCase: Acetyl-CoA carboxylase carboxyl transferase
ACP: Acyl-carrier protein
ACSL: Long-chain acyl CoA synthetase
BLAST: Basic local alignment search tool
COG: Clusters of orthologous groups
DAF: Days after flowering
DAG: Diacylglycerol
DEG: Differential expression gene
DGAT1: Diacylglycerol O-acyltransferase 1
EAR: Cnoyl-ACP reductase
ER: Endoplasmic reticulum
FA: Fatty acid
FAD2/6: Omega-6 FA desaturase
FATA: fatty acyl-ACP thioesterase A
FATB: Fatty acyl-ACP thioesterase B
G3P: Glycerol-3-phosphate
GO: Gene ontology
GPAT: sn-1 G3P acyltransferase
KAR: 3-ketoacyl ACP reductase
KASII: 3-ketoacyl ACP synthase II
KASIII: 3-ketoacyl ACP synthase II
KEGG: Kyoto encyclopedia of genes and genomes
Lipid substrates are abbreviated: 16:0, palmitic acid
LPAAT: Lysophosphatidyl acyltransferase
LPCAT: Lysophosphatidylcholine acyltransferase
MAT: Malonyl-CoA-ACP transacylase
Nr: NCBI non-redundant protein
PA: Phosphatidic acid
PC: Phosphatidylcholine
PDAT1: Phospholipid, diacylglycerol acyltransferase1
PLA2: Phospholipase A2
qRT-PCR: quantitative real-time PCR
RNA-Seq: RNA sequencing
SAD: 18:0-ACP desaturase
SRA: Short read archive
TAG: Triacylglycerol
TCA cycle: Tricarboxylic acid cycle
