# Supplementary figures and images for: Transcriptome analysis revealed the dynamic oil accumulation in Symplocos paniculata fruit
Source: BMC Genomics. 2016 Nov 16;17:929. doi: 10.1186/s12864-016-3275-0 (PMC5112726; doi:10.1186/s12864-016-3275-0)

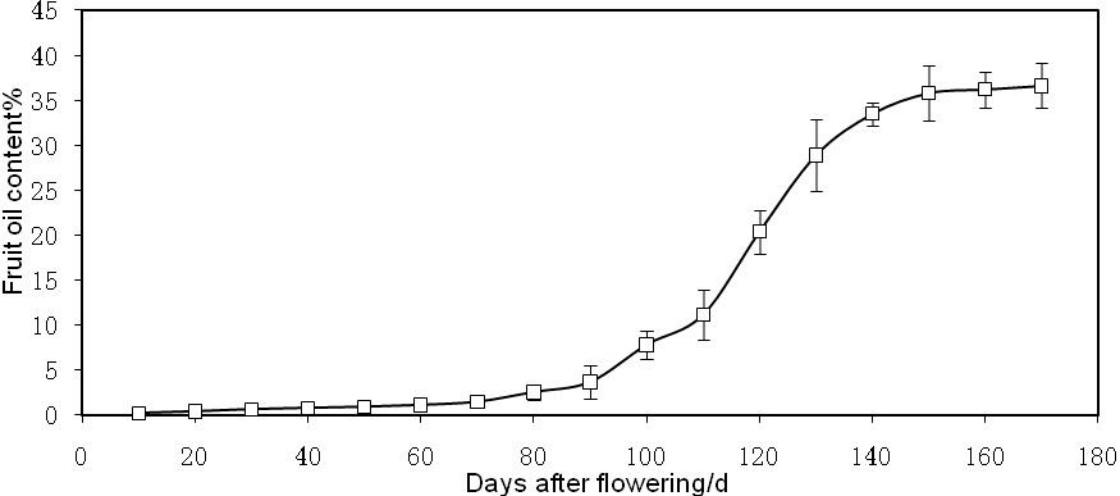

Supplement: Additional file 1: Figure S1. — Dynamic change of fruit oil content during the fruit development. (PDF 33 kb) [file 12864_2016_3275_MOESM1_ESM.pdf]

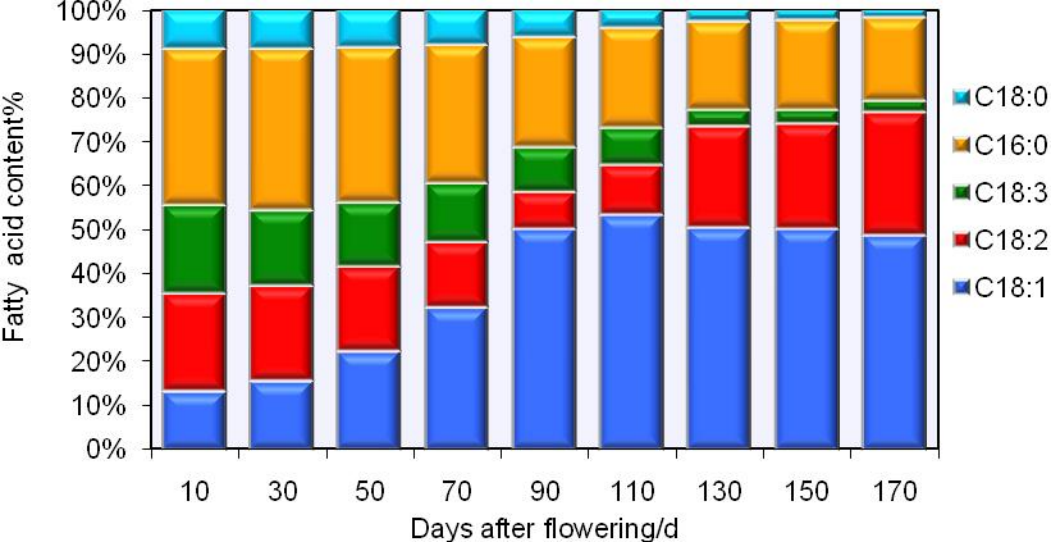

Supplement: Additional file 2: Figure S2. — Dynamic change of the main fatty acids in fruit oil during the fruit development. (PDF 59 kb) [file 12864_2016_3275_MOESM2_ESM.pdf]

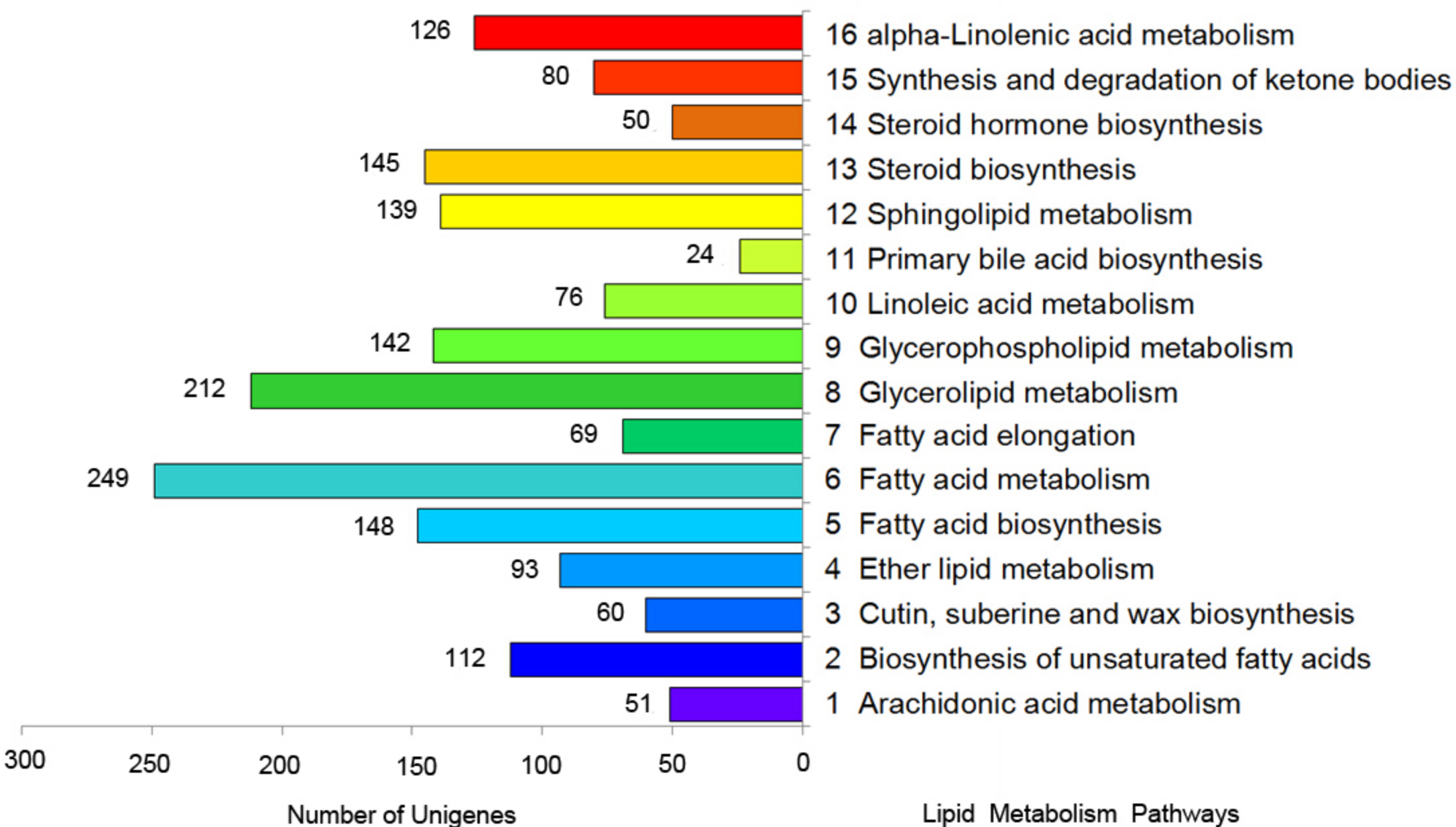

Supplement: Additional file 7: Figure S3. — Distribution of unigenes classified to 16 lipid metabolism pathways. (PDF 384 kb) [file 12864_2016_3275_MOESM7_ESM.pdf]

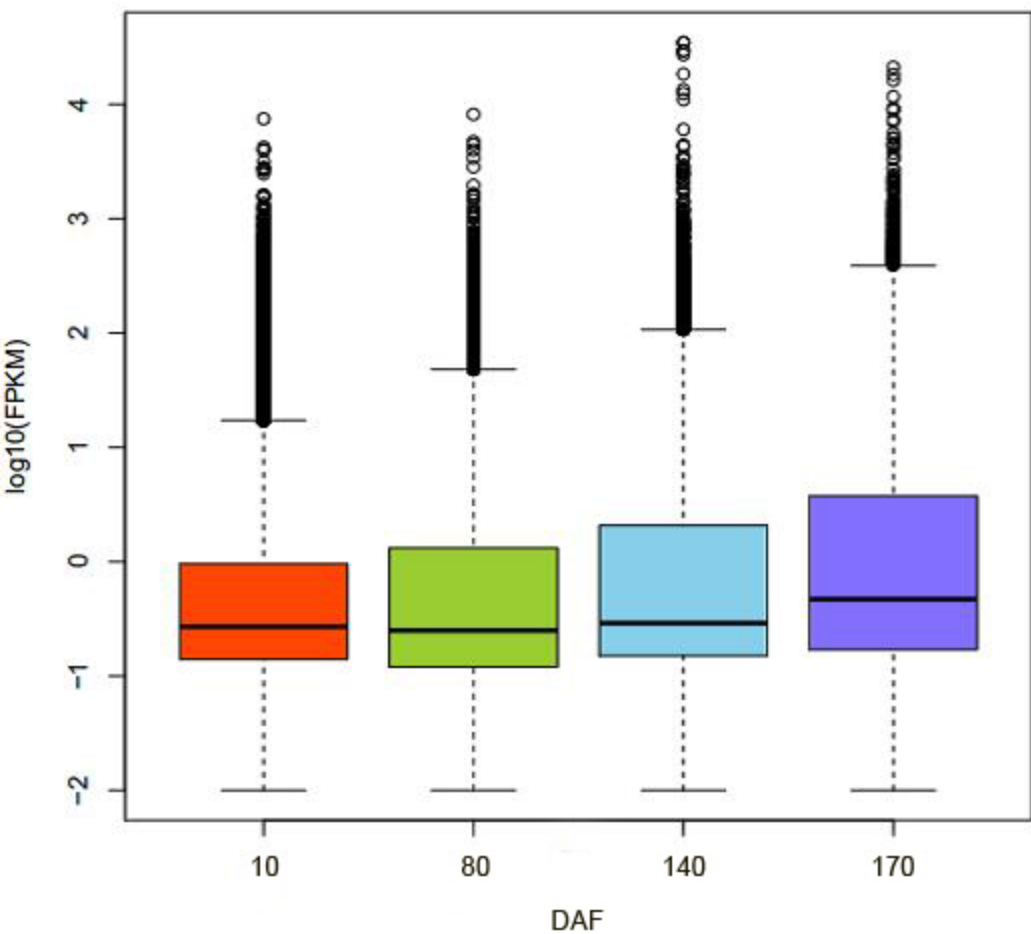

Supplement: Additional file 9: Figure S4. — The RPKM distribution of unigenes. (PDF 72 kb) [file 12864_2016_3275_MOESM9_ESM.pdf]
